# Human lagochilascariasis—A rare helminthic disease

**DOI:** 10.1371/journal.pntd.0005510

**Published:** 2017-06-22

**Authors:** Dulcinea Maria Barbosa Campos, Alverne Passos Barbosa, Jayrson Araújo de Oliveira, Giovana Galvão Tavares, Pedro Vitor Lemos Cravo, Alejandro Luquetti Ostermayer

**Affiliations:** 1 Programa de Pós-Graduação em Sociedade Tecnologia e Meio Ambiente e Curso de Farmácia, Centro Universitário de Anápolis–UniEVANGÉLICA, Anápolis, Goiás, Brazil; 2 Instituto de Patologia Tropical e Saúde Pública, Universidade Federal de Goiás, Goiânia, Goiás, Brazil; 3 Instituto de Ciências Biológicas, Universidade Federal de Goiás, Goiânia, Goiás, Brazil; 4 Instituto de Higiene e Medicina Tropical, Universidade Nova de Lisboa, Lisboa, Portugal; Universidad San Francisco de Quito, ECUADOR

## Abstract

Lagochilascariasis is a parasitic disease caused by a helminth of the order Ascaroidea, genus *Lagochilascaris* that comprises 6 species, among which only *Lagochilascaris minor* Leiper, 1909, is implicated in the human form of the disease. It is remarkable that the majority of cases of human lagochilascariasis in the Americas have been reported in Brazil. The natural definitive hosts of this parasite seem to be wild felines and canines. Lagochilascariasis is mostly a chronic human disease that can persist for several years, in which the parasite burrows into the subcutaneous tissues of the neck, paranasal sinuses, and mastoid. *L*. *minor* exhibits remarkable ability to migrate through the tissues of its hosts, destroying even bone tissue. Fatal cases have been described in which the parasite was found in the lungs or central nervous system. Treatment is often palliative, with recurrence of lesions. This paper summarizes the main features of the disease and its etiologic agent, including prevalence, life cycle, clinical course, and treatment.

## Introduction

Robert T. Leiper, 1909, a helminthologist at the London School of Tropical Medicine and Hygiene who received specimens of a Nematoda recovered from subcutaneous abscesses of 2 patients from Trinidad (off the coast of South America), was the first to describe *Lagochilascaris minor*. At that time, the digestive tract of a carnivore was suggested as the probable habitat for *L*. *minor*. He proposed that the finding of parasites in subcutaneous abscesses in humans from Trinidad was remarkable evidence that an animal, other than a human, could be the definitive host for this helminth [1]. Since then, more than 100 cases of purulent abscesses in humans have been reported in different countries in the Americas. *L*. *minor* has been found in subcutaneous abscesses in the cervical region [1–5], mastoid [4–9], rhino-oropharynx [4,5,9,10], tonsils [4,5,11,12], auditory meatus [4,5,10,13], nasal sinuses [9,13–15], lungs [4,5,16], central nervous system [6,13,17]_,_ sacral region [4], eyes [10,18], and dental alveoli [5,9,19] of humans. Different stages of the life cycle (eggs, larvae, and adult worm) have also been found [5,14,20,21]. It is remarkable that all these organs differ from the digestive tract, the normal habitat of all other ascarides. Corroborating Leiper’s, 1909, assumption, a wild felid, *Puma concolor*, naturally infected by *L*. *minor* was recently found in Mexico [22].

## Methods

A literature review on the subject was conducted, using publications from this group and other scientific articles published in indexed journals in the Latin American and Caribbean Health Sciences database (LILACS), Scientific Electronic Library Online (SciELO), and *Index Medicus* (MEDLINE). The authors of this manuscript did not participate in any clinical activity involving humans. Human involvement was limited to the contribution of parasitological diagnosis in case reports, whose photographs were authorized by the patients. From the standpoint of experiments using animals, based on Brazil’s Federal Law No. 11,794 of 8 October 2008, the projects of this group were carried according to the protocol approved by the Ethics Committee on Animal and Human Medical Research of the Clinical Hospital at the Federal University of Goiás and, subsequently, by the Ethics Committee on Animal Use (CEUA) of the Federal University of Goiás, which is subordinate to the National Council for the Control of Animal Experimentation (CONCEA).

### Geographical distribution and prevalence

**Fig 1:**
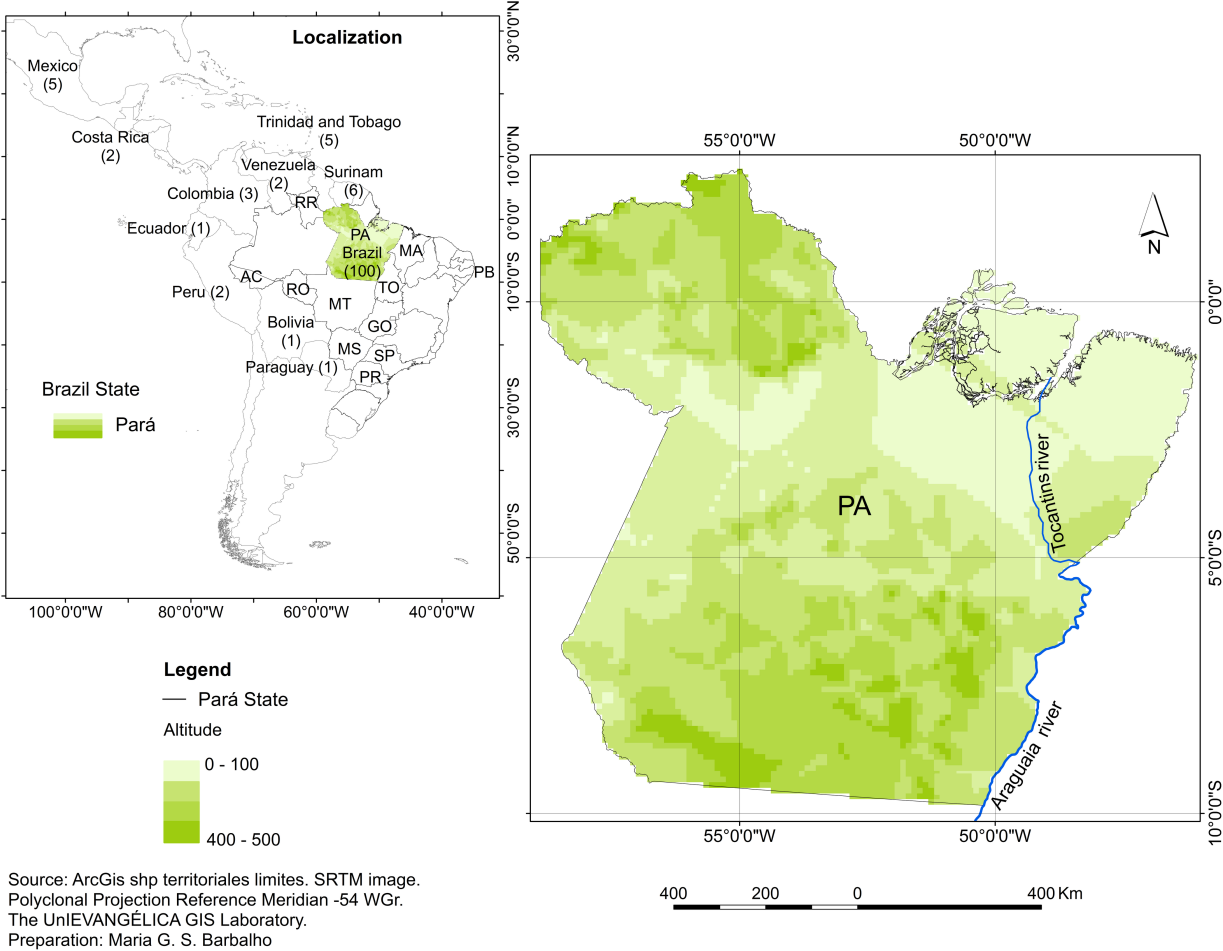
Geographical distribution of human lagochilascariasis in the Americas.

Human lagochilascariasis has been recorded in Trinidad and Tobago [1,2,18,23], Surinam [14,20,24,25], Mexico [9,26], Costa Rica [27], Venezuela [6,15,28], Colombia [11,12], Bolivia [29], Ecuador [30], Paraguay [7], Brazil [3–5,10,13,16,21,31–35] and Peru [36] (Fig 1). As for its geographic distribution by country, the highest concentration is found in Brazil, representing 78.1% (100/128) of the total number of cases reported in the literature [36]. Among the cases in Brazil, the majority (60%, 60/100) were recorded in the state of Pará, followed by Rondônia, Tocantins, Mato Grosso, Acre, Roraima, São Paulo, and Paraná (Fig 1). It should be noted that only 1 case of human lagochilascariasis has been recorded in each of the following states: Maranhão, Paraíba, Mato Grosso do Sul, and Goiás [36].

### The disease in humans

**Fig 2:**
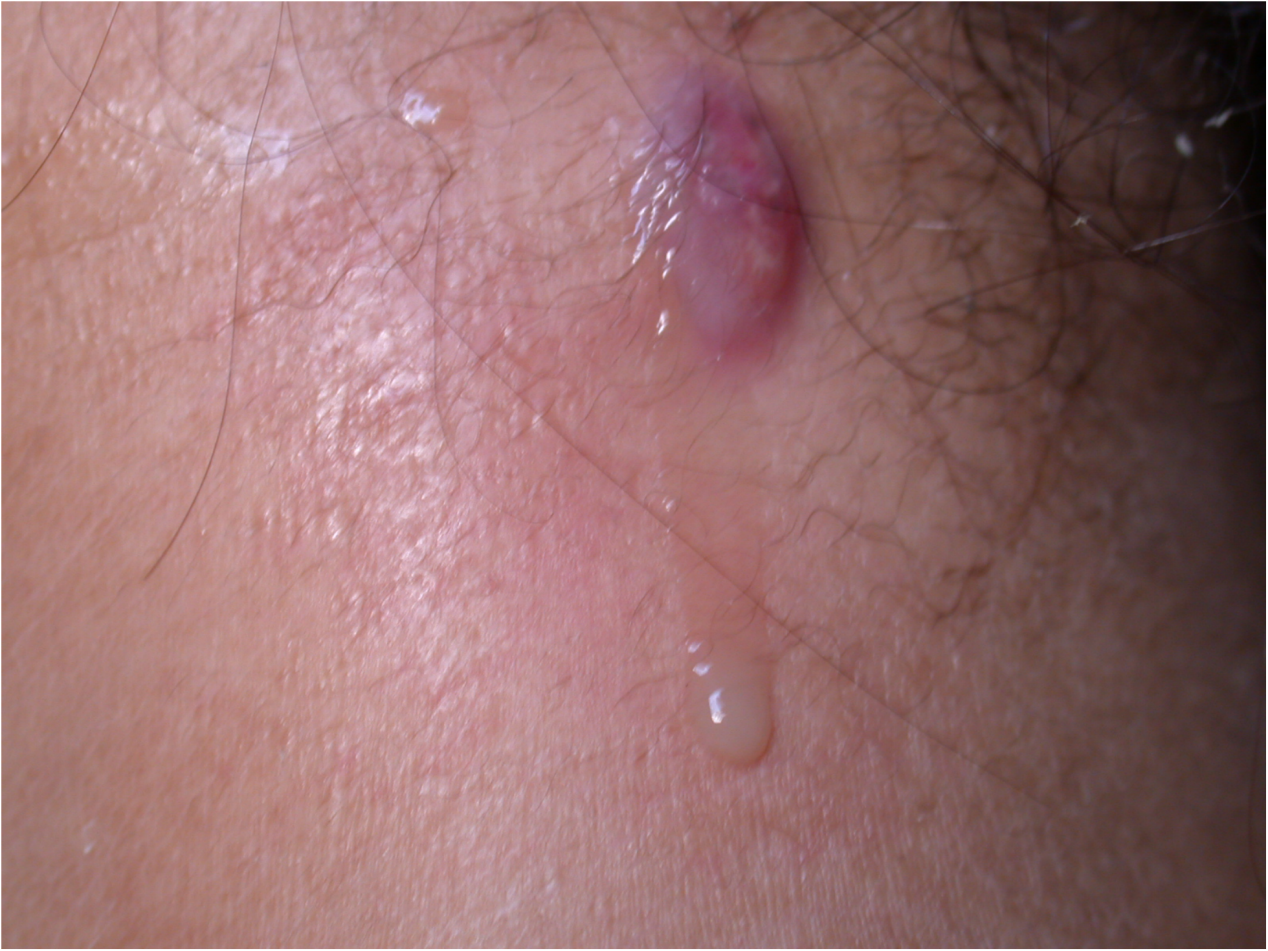
Cervical lesion draining pus in a patient infected with *Lagochilascaris minor*.

**Fig 3:**
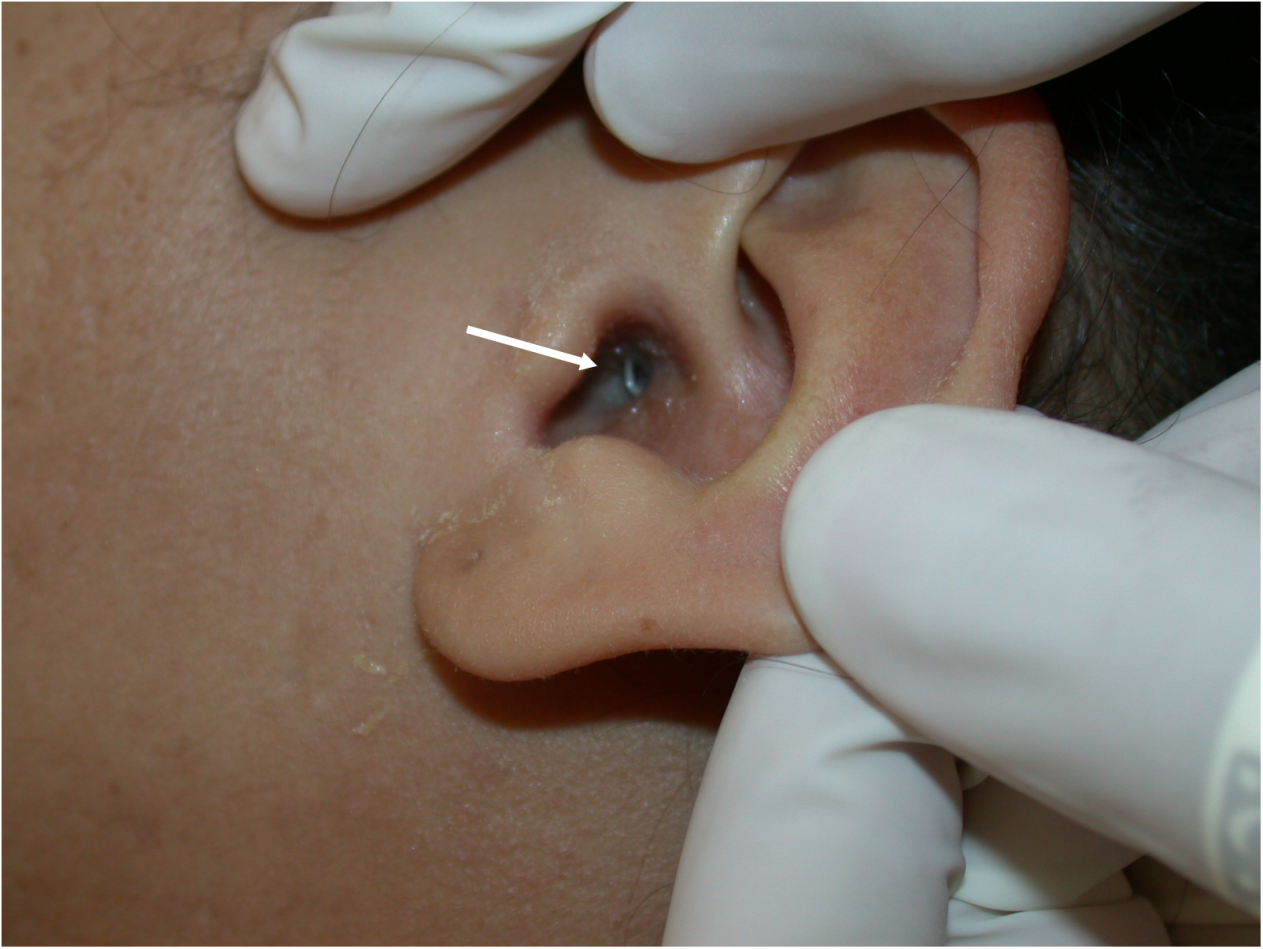
Adult worm of *Lagochilascaris minor* migrating from the mastoid to the external auditory meatus.

Lagochilascariasis in humans is often misdiagnosed as an abscess of common causes, mainly of bacterial etiology. It presents more often as a subcutaneous abscess on the neck (Fig 2), middle ear (Fig 3), mastoid, tonsils, and nasal sinuses, which physicians in countries in the Neotropical region should check.

Other common localizations of the parasite are the central nervous system, lungs, sacral region, eyeballs, and dental alveoli [21,34,35]. Usually, the patient seeks medical help after several days of discomfort and is examined by several physicians, showing poor response to antibiotics [9]. The lesion usually evolves slowly over weeks or months. Some patients report the formation of a tumor in the neck, initially small, without fistula and with no pain [4,5,21,35]. As the disease progresses, the tumorous lesion becomes painful, with or without spontaneous fistula, with drainage of a serous purulent exudate, generally containing small whitish worms, eggs, and larvae [4,5,21,35]. The size of the tumorous lesion may vary from 5 to 12 cm and is usually found in the cervical region, with the aspect of a pseudocyst, nodule, or abscess [8,37]. It is usually a painful lesion with a hard consistency and undefined limits. The migration of the parasite through the host’s tissues originates secondary lesions that can be located close to or very far from the initial abscess [4,5].

In lagochilascariasis, both symptoms and magnitude of the disease depend upon the location of the parasite, the parasite load, and certainly the immune response by the host, who may be able to control the pathogenic processes, as well as limit the establishment of new lesions [5].

Some reports refer to chronic abscesses of the auditory meatus (with purulent exudate for 1 or 2 years) and painful tumor in the mastoid [7,9], which may progress to neurological involvement [13,17]. Otalgia and purulent otorrhea have been recorded. Otoscopy in the right ear shows retroauricular swelling, polyps in the external ear canal, and fistula with drainage of pus [38]. In cases of otitis and mastoiditis, the X-ray examination shows extensive areas of osteolysis in the mastoid region [13]. The osteolytic capacity of this parasite has also been reported in the destructive lesion of the sacral bone, as well as of the 4^th^ and 5^th^ lumbar vertebrae and soft tissues adjacent to the sacrum [4]. In other cases, lesions in the middle ear and mastoid extend to the base of the skull, evolving into extradural abscesses and instances of neck stiffness. The preceding phase may be characterized by a history of ear drumming, intense headache with the pain radiating to the hemiface, and, finally, elimination of worms through the oral cavity [13,32]. Other authors have reported finding only 1 nodule with fistula in the neck in patients at the time of diagnosis and elimination of the adult worms through the oral cavity and auditory meatus a few months later [4,5,11].

Neurological involvement due to *L*. *minor* infection may develop in the absence of lesions in the neck, with clinical manifestations such as seizures, headache, paresthesia, motor alterations, cerebellar ataxia, and mental confusion [17]. Vomiting, papilledema, and facial paralysis have been described as well [13]. A fatal case of encephalopathy due to *L*. *minor* with a subacute, progressive disease characterized by headache, stupor, and coma has been reported [17]. In this patient, death occurred 3 months after the beginning of the illness. Neuropathological examination revealed diffuse foci of necrosis of the cerebral hemispheres and cerebellum and presence of the nematode in the parenchyma and in the cisterns at the base of the brain [17].

Bilateral bronchopneumonia, with hundreds of abscesses measuring 2–5 mm scattered throughout the pulmonary parenchyma, has been reported [16]. Microscopically, in lungs, in each low-power field, at least 1 abscess was visible, the majority of them containing fourth stage larvae, young adults, or mature adult worms. In addition to the unusualness of its location in the lungs, all the evolutionary stages of the worm were found in the affected organ, i.e., eggs, larvae, and adult worms [16], a fact that characterizes the autoinfecting cycle of the parasite. There are reports of pneumonia occurring with fever and dyspnea, which progressed to cyanosis, respiratory insufficiency, and death less than 3 months after the onset of symptoms [16].

Chronic tonsillitis occurring with the sensation of worms moving through the throat, elimination of worms through the mouth, sensation of ingesting worms, headache, hearing loss, and overall debility have been observed in infections of tonsils and the middle ear [11].

An important clinical issue is the distinction between otitis, mastoiditis, sinusitis, and tonsillitis caused by *L*. *minor* infection and other related diseases. Clinicians, particularly otorhinolaryngologists and neurologists, working in Neotropical regions should be attentive to information about the discharge of adult worms through nasal sinuses, mouth, or auditory meatus [5]. The proteolytic enzymes in *L*. *minor* can facilitate its migration through the host’s tissues by hydrolyzing collagens of the extracellular matrix [39].

### The life cycle

An experimental model involving mice and domestic cats was described in an attempt to unravel the life cycle of *L*. *minor* [21,40]. Mice act as intermediate hosts and domestic cats as definitive hosts of this helminth [21,40]. In that study, eggs recovered from human lesions were stored in 1% formaldehyde at room temperature (20–33°C) for a period of approximately 30 days in order to obtain the third stage (infecting stage) larvae [21,34,41,42].

**Fig 4:**
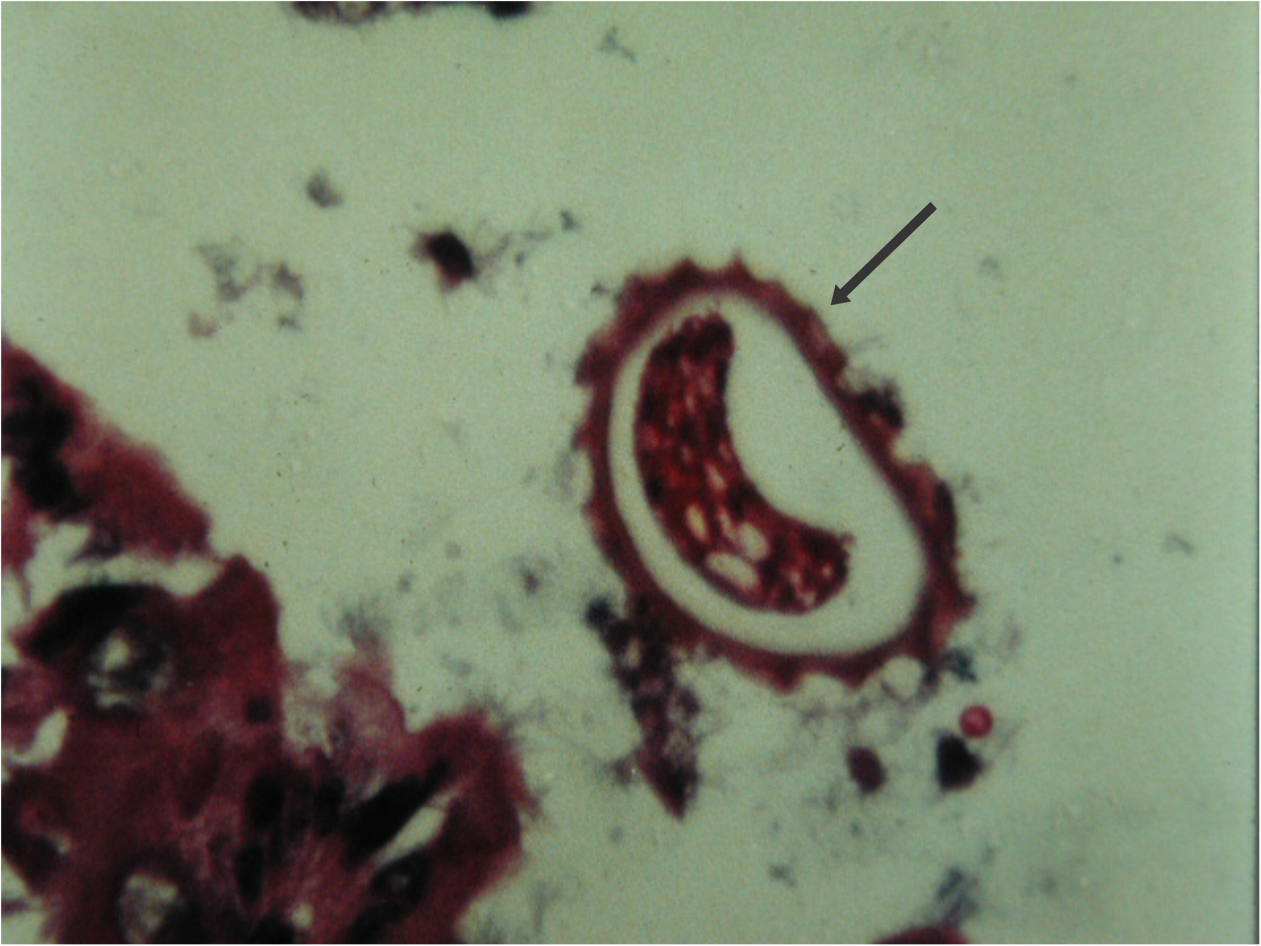
*Lagochilascaris minor* infecting egg in the intestinal lumen of an experimentally infected mouse.

In the intermediate host (mouse) orally inoculated with infecting eggs, larvae hatched in the later part of the small intestine and cecum 4 to 6 hours after infection [21,40–42] (Fig 4).

**Fig 5:**
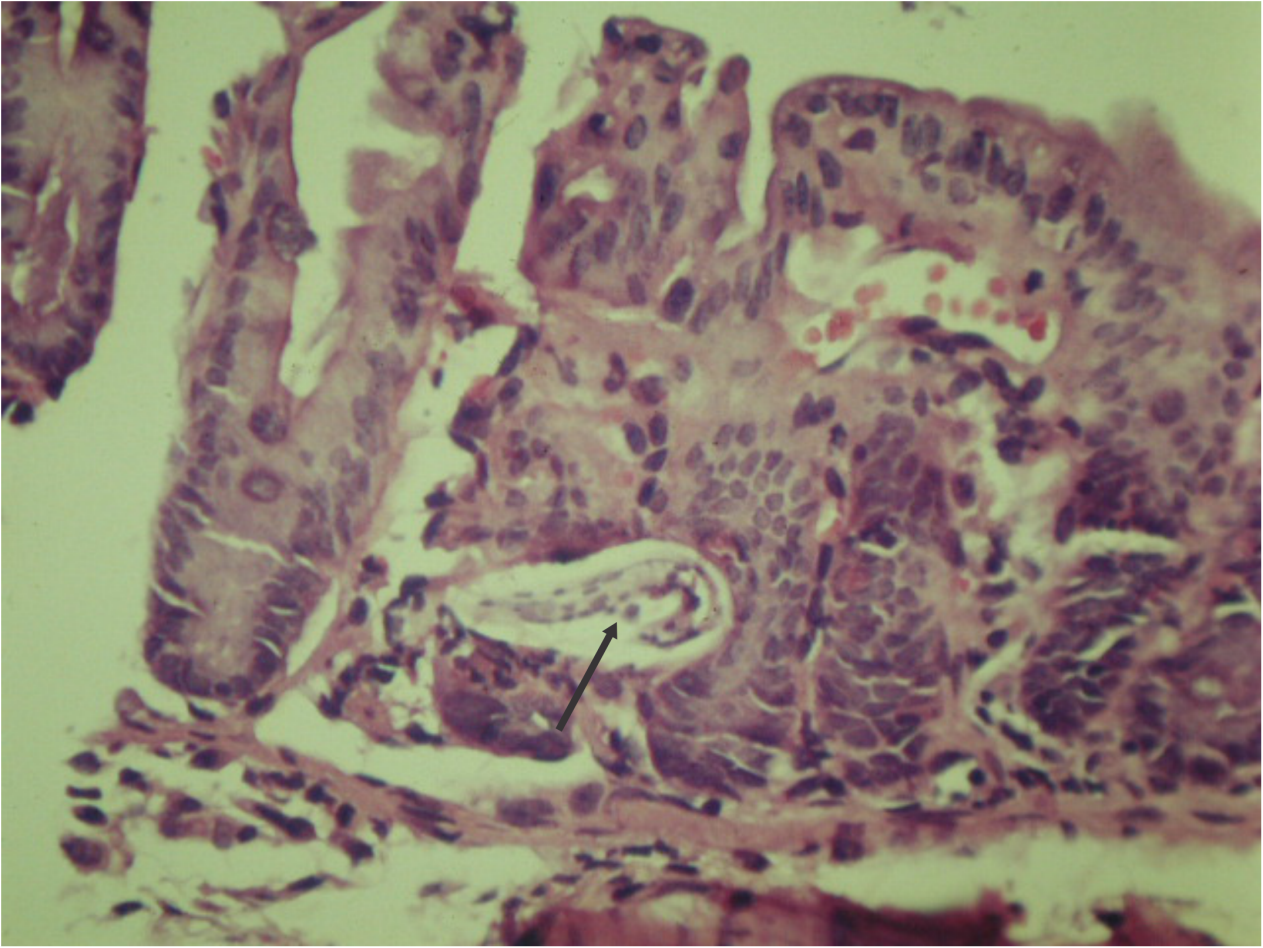
Third stage larvae of the *Lagochilascaris minor* crossing the cecal mucosa of an experimentally infected mouse.

Approximately 6 h after inoculation, early third stage larvae were first observed passing through the mucosa in distal portions of the small intestine and cecal mucosa (Fig 5).

**Fig 6:**
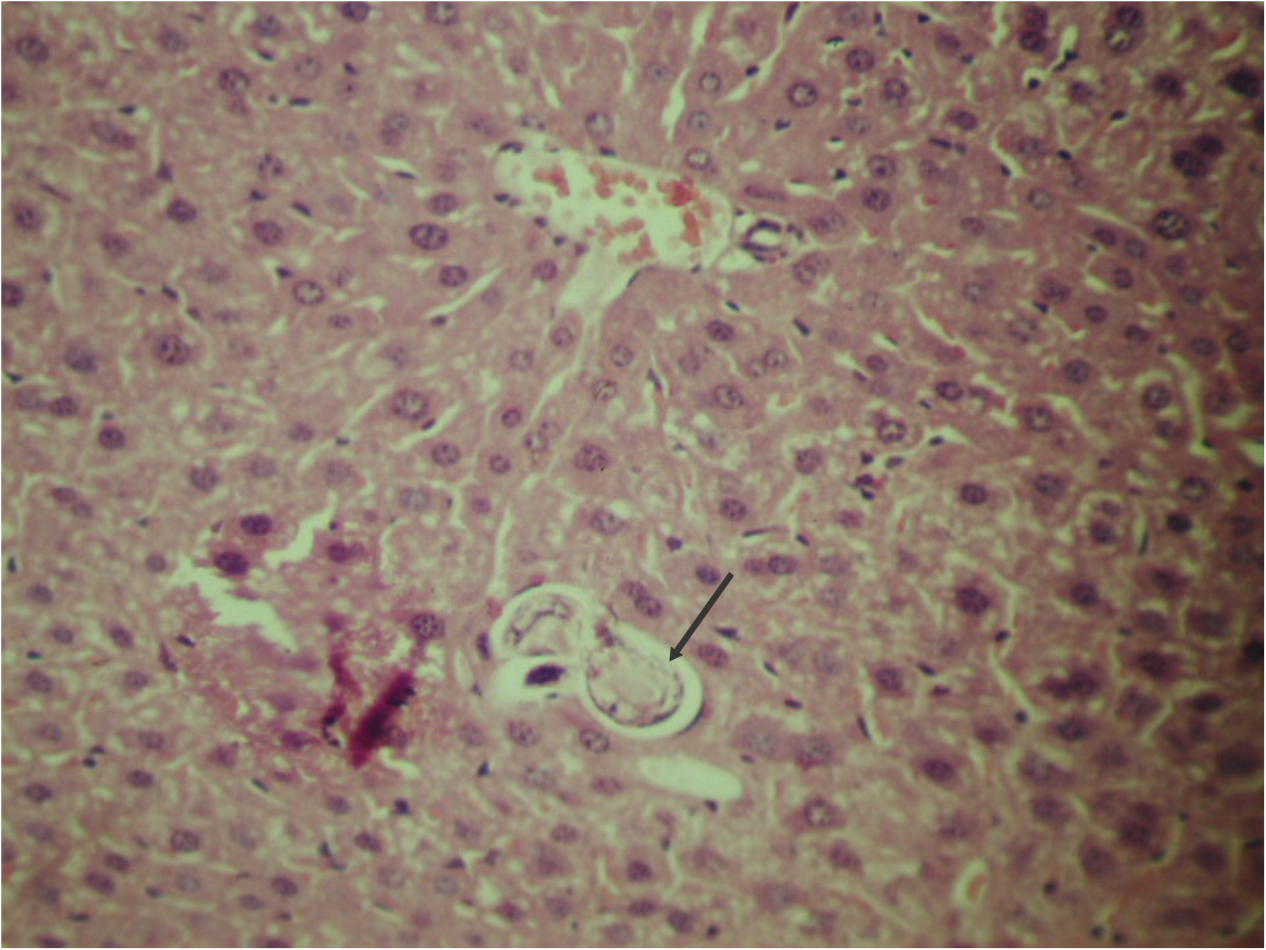
Third stage larva in the hepatic parenchyma of an experimentally infected mouse.

Following the hatching period, larvae were found inside lymphatic vessels and the hepatic portal vein, reaching the hepatic parenchyma (Fig 6) and lungs in 24–48 hours [21,40–42].

**Fig 7:**
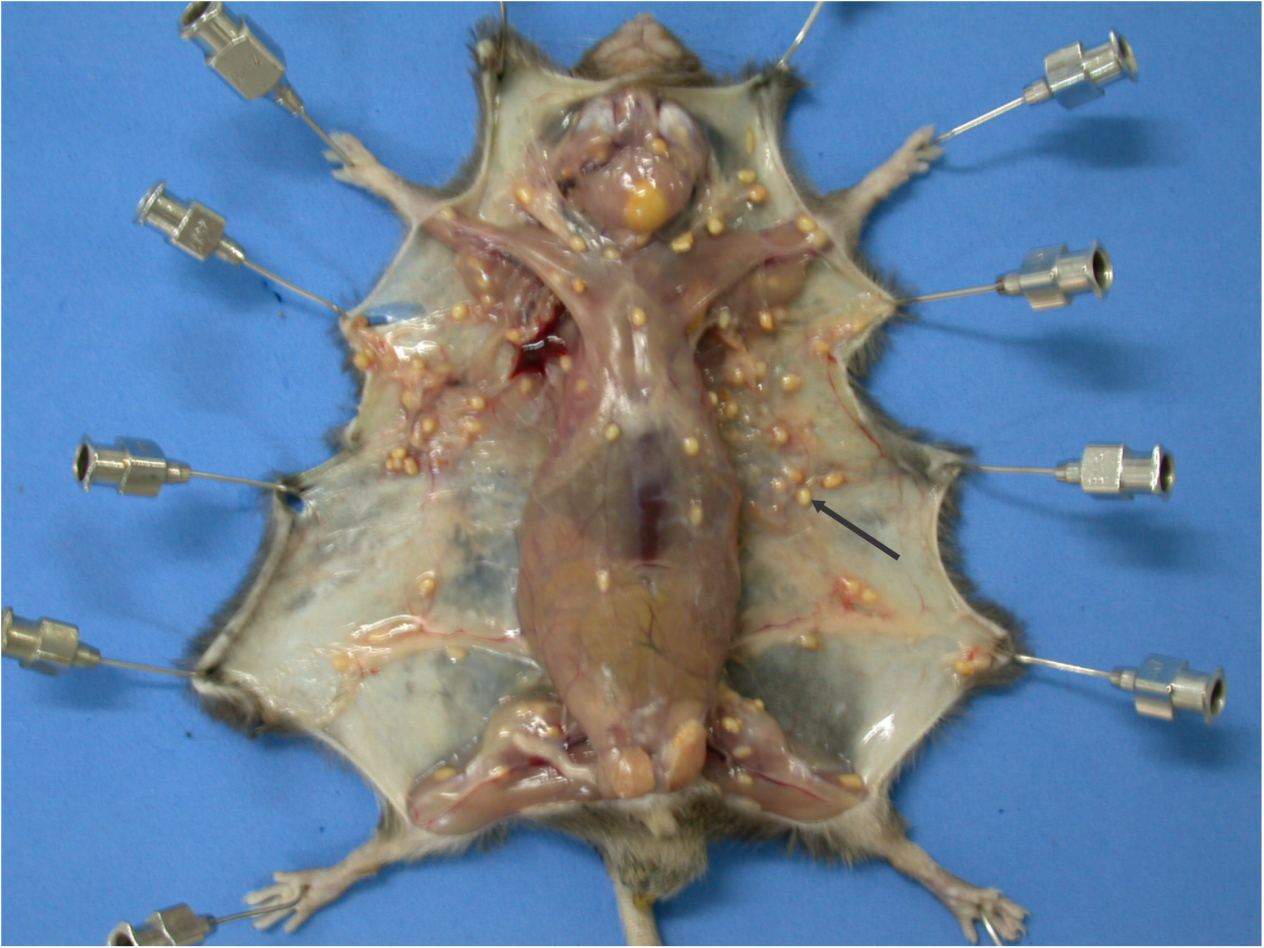
Mouse infected with *Lagochilascaris minor* eggs. Granulomatous nodules containing third stage larvae in the muscles and cellular subcutaneous tissue.

After migration, larvae encyst in skeletal muscles and subcutaneous tissues [21,40–42] (Fig 7). Nodules were distributed irregularly in the muscles of the cervical, thoracic, abdominal, lumbar, axillary, and paw regions of the mice [21,41]. Encysted larvae were also found in the liver, lungs, and heart. Adult worms may also sometimes be found inside nodules of experimentally inoculated mice [41].

**Fig 8:**
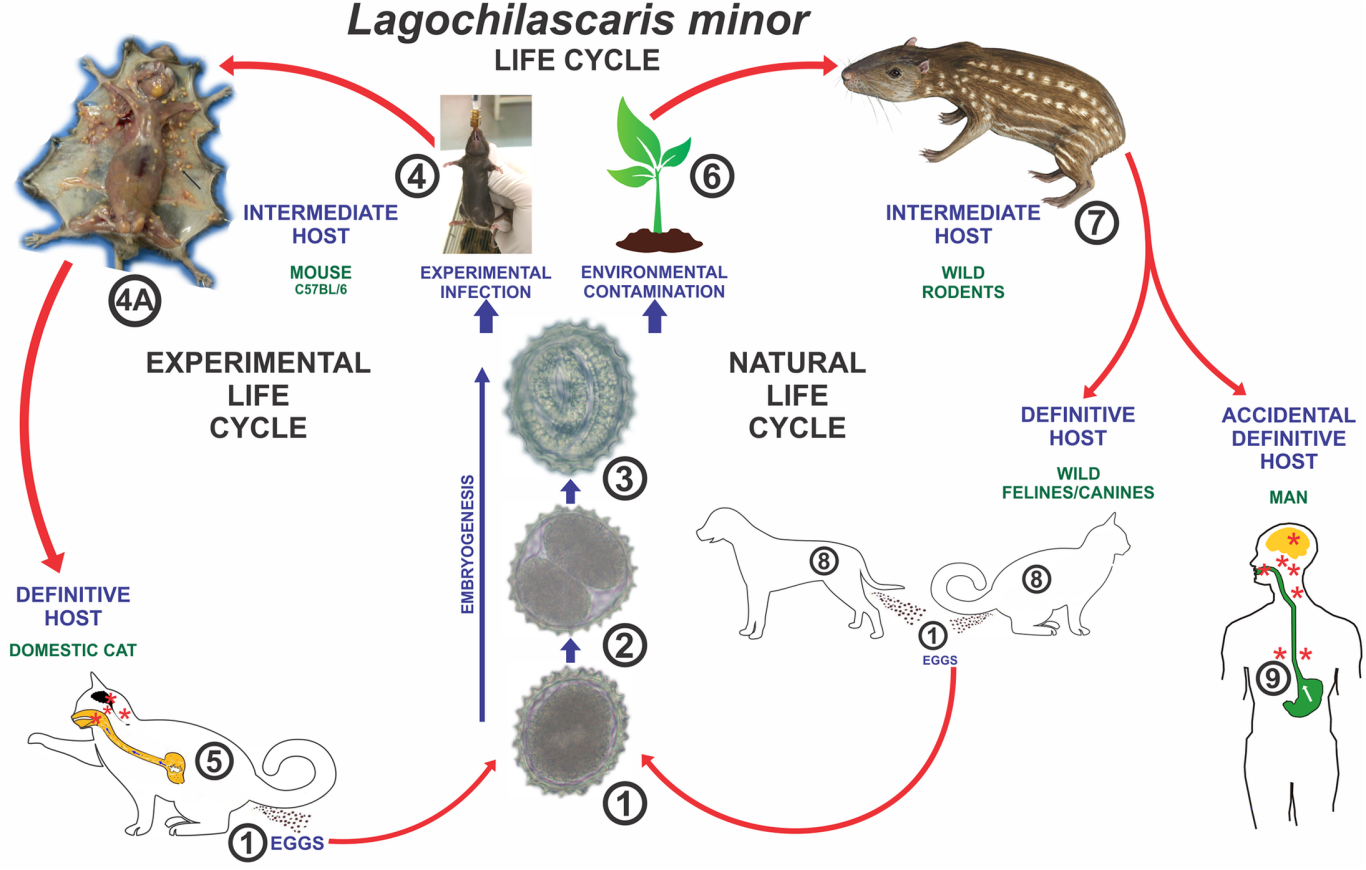
Life cycle of *Lagochilascaris minor*. Parasite eggs are eliminated from the host organism through feces (1), undergo division (2), and develop into the infecting stage (3). The infecting egg may be either orally inoculated into the mouse (4) or contaminate the environment (6). In experimental infection, granulomatous nodules containing third stage larvae are observed in the muscles and subcutaneous tissue of a mouse infected with the helminth (4A). Experimental definitive hosts are infected through ingestion of intermediate hosts containing third stage-encysted larvae (5). Once in the environment (6), infecting eggs are ingested by wild rodents (7). Wild felines/canines ingest intermediate hosts containing third stage larvae and eliminate parasite eggs in the environment through feces (8). Human infection originates from the ingestion of uncooked or partially cooked meat of wild rodents containing encysted larvae (9).

When cats (definitive hosts) ingest infecting eggs *per os*, the parasites do not reach sexual maturity [21,34,40,41]. However, when cats are fed with mouse carcasses infected with third stage larvae, larval hatching from cysts occurs in the stomach [21,34,40]. After hatching, larvae migrate to upper regions of the digestive tract, reaching the adult stage in tissues of rhino and oropharynx (tonsils and soft palate, including unilateral or bilateral lesions), nasal sinuses, middle ear, mastoid, cervical lymph nodes, lungs, and brain [21,34,40]. After 3 hours of inoculation, third stage larvae are found almost exclusively in the stomach, although some have also been found in the esophagus, rhino, and oropharynx [21]. At 6 hours post-inoculation, third stage larvae are predominately found in tissues of rhino and oropharynx, and only a few larvae are left in the stomach. Moreover, fourth stage larvae are observed from 2 to 8 days, while adult worms can be seen from 9 to 20 days post-inoculation. Both the 3^rd^ and 4^th^ ecdysis can occur in any of the above-mentioned locations but not the stomach [21] (Fig 8). The outcome of experimental infection in cats is the formation of tumorous masses and tunnels through different tissues of the host as a result of *L*. *minor* migration. Eggs can be found either directly in the lesions or in the host’s feces when abscesses in rhino or oropharynx fistulate towards the digestive tract lumen [21,33,34,40]. The occurrence of autoinfecting cycles has been reported in both humans [4–6,16,21,33] and cats [4,21,35]. Eggs, mostly embryonated third stage larvae, and various developmental stages of the worm were found in cervical nodules of a patient from Paragominas (PA, Brazil), thus proving the existence of human autoinfection [33]. The finding of these evolutive stages confirms the ability of *L*. *minor* to reproduce in human tissues (autoinfection) and provides an explanation for the long duration of the infection in humans [33]. At necropsy of a cat experimentally infected, Campos et al. [21] observed the occurrence of the auto-infecting cycle of *L*. *minor*. Eggs with 2, 4, and 8 blastomeres, eggs containing larvae, and third stage larvae were found in the tissues of the neck and lungs at necropsy on day 43 post-infection.

### Transmission mechanisms

After infecting wild rodents, namely *Dasyprocta agouti* (agouti), *Calomys callosus*, and *Cavia porcellus* (guinea pig) with *L*. *minor* eggs, the formation of nodules containing third stage larvae was observed in skeletal muscle, subcutaneous tissues, adipose tissue, and viscera [34]. Adult worms found in abscesses in the cervical region, rhino, and oropharynx were recovered from cats fed with carcasses of infected rodents [21,34].

The findings of Campos et al. [21] and Paçô et al. [34] corroborated the hypotheses of Smith et al. [43], who suggested that human infection by *L*. *minor* originates from the ingestion of uncooked or partially cooked meat of wild animals containing encysted larvae. Campos et al. [21] suggested that larvae kept in tissues of rodents could hatch in the human stomach and, from there, migrate towards the upper regions of the digestive tube and neighboring tissues, such as tonsils, middle ear, nasal sinuses, mastoid, and all the other locations where worms have been found. It is also assumed that larval hatching from nodules enables larvae to reach the upper regions of the digestive tract, and then the tissues of the pharynx, without necessarily undergoing a cardiopulmonary cycle. Campos et al. [21] and Campos & Barbosa [5] suggested that some components of the digestive tract of carnivores hinder *L*. *minor* third stage larva inside the egg. It has also been suggested that the passage of the helminth through the intermediate host body is fundamental for the parasite to acquire further resistance, enabling its later development in the definitive host. Therefore, the intermediate host plays a fundamental role in worm development [5,21]. After the worm reaches the adult stage in human tissues, the autoinfecting cycle may initiate [5].

The Neotropical region corresponds to Central America, South America, and parts of both Mexico and the United States of America. It presents a high degree of biodiversity because it encompasses varied ecosystems such as the Amazon rainforest and Magellanic subpolar forests [44]. If the digestive tract of carnivores (wild felines/canines) is a normal habitat for the helminth, eggs eliminated by feces could contaminate the soil (Fig 8). Wild rodents, intermediate hosts or paratenic hosts, become exposed to infection by ingesting embryonated eggs in the environment. Consequently, wild rodents could play an important role in the chain of epidemiological transmission of this parasite [5,21].

### Diagnosis

Clinical diagnosis is rarely performed in the initial stages of the disease. Infected individuals only seek medical assistance in the advanced stages of the disease [5,9,21]. The aspect of the cervical lesions involves differential diagnosis with pyogenic adenitis, actinomycosis, paracoccidioidomycosis, ganglionar tuberculosis, and leishmaniasis [5,19,35]. Clinical diagnosis is remarkably difficult when involvement of the central nervous system, lungs, and even rhino and oropharynx is present and also if no visible tumor in the cervical, retroauricular, and mastoid regions is observed. These cases are often only confirmed at autopsy [5].

Parasitological diagnosis is based on the finding of the parasite obtained from the lesion. Adult worms and larvae should be fixed and stained. When *L*. *minor* is located in tissues of rhino and oropharynx, the formation of fistula may allow the migration of eggs to the intestinal lumen. The similarity of eggs of *L*. *minor* with those of *Ascaris lumbricoides* requires the differentiation of both species. Eggs of *L*. *minor* may be found not only in the cervical region, mastoid, and feces but also in exudate of the auricular meatus, paranasal sinuses, and pulmonary secretion [5,6,35].

The contents of abscesses of retroauricular and cervical regions, as well as fragments of other biopsied tissues, could be examined by thin-layer histopathology and hematoxylin-eosin staining [37]. Eggs or fragments of the worm, as well as larvae inside granulomas or micro-abscesses, are visible through microscopy [37,42]. Other procedures such as rhinoscopy, otoscopy, transnasal stereotactic biopsy, and imaging methods such as computerized tomography and magnetic resonance may be useful in diagnosis [38].

There are no standardized methods available for the immunological diagnosis of lagochilascariasis.

### Treatment

Several drugs, such as benzimidazole derivatives, ivermectin, and diethylcarbamazine (Table 1), have been used in the treatment of lagochilascariasis [9,10]. Treatment generally starts with thiabendazole, followed by diethylcarbamazine or followed by mebendazole and, finally, levamisole [11,32,33]. After the entire therapeutic arsenal is used against lagochilascariasis, a common conclusion is that it is difficult to achieve complete remission or cure of this disease. Following treatment with levamisole, hundreds of specimens of *L*. *minor* are eliminated, and the lesion heals, a phenomenon commonly mistaken for a cure. However, the apparent clinical cure is usually followed by relapses to previous conditions [5]. Complete cure in human lagochilascariasis is infrequent. Treatment interruption will lead to new tumor formation close to or far from the initial lesion, and consequently, the infected tissue becomes full of healing scars. Female adult worms present on the tissues produce eggs, and the resulting larvae are able to originate new adult worms, starting a new cycle. This is called the autoinfecting cycle of *L*. *minor* [5,16,33]. Acute relapses of the disease are due to egg embryogenesis and the development of all other forms of the parasite, completing its life cycle and reducing the chances of therapeutic protocol being effective. An ideal drug should be effective against eggs, larvae, and adult worms and should also be able to prevent egg embryogenesis [5,6]. The lack of such a drug implies the use of long and ineffective treatments. It is assumed that thiabendazole and levamisole are both potent drugs against adult worms and are probably effective against larvae as well. Often, however, both drugs are ineffective against eggs. Consequently, eggs can keep on developing and ultimately lead to larval hatching, giving rise to adult worms and originating new lesions [5,6,14,20,21,25]. However, the combination of prolonged drug use and surgical removal of the mass seems to lead to a favorable outcome in some cases [45]. Campos et al. [46] described a patient from Pará, Brazil with a chronic infection of *L*. *minor* who was resistant to treatment with dietilcarbamazine, levamisole, albendazole and ivermectin. Authors emphasized that all evolutive stages of the helminth were present in the lesion, a finding that characterizes the existence of an autoinfecting cycle [46].

**Table 1:** Drugs used against lagochilascariasis. Therapeutic protocols in 20 patients infected by *Lagochilascaris minor*.

PATIENT N°	ABSCESS	DRUGS	DOSES	EVOLUTION	REF
1	CR and NS	diethylcarbamazine	Three 50 mg tablets three times a day (1,000 tablets)	Chronic disease	[2]
2	CR	thiabendazole	500 mg twice a day for 3 successive days	Patient died	[14]
3	CR		50 mg/Kg of body weight a day for 5 days	No follow-up	[20]
4	Ms, ME and CNS		50 mg/Kg of body weight a day for 2 days	Patient died	[13]
5	Ms, ME, CR and CNS		2,000 mg/24 h	Patient died	[6]
6	CR	levamisole	150 mg a day for 3 days	Probable cure	[3]
7	SMR	pyrantel pamoate	700 mg for 5 days	No follow-up	[11]
8	CR	levamisole	150 mg a day for 4 weeks	Probable cure	[50]
9	NS	albendazole	400 mg (single dose)	No cure	[9]
10	Ms		400 mg once a day for 30 days	No follow-up	[9]
11	CR	thiabendazole	500 mg twice a day for 3 days	No cure	[32]
		
		diethylcarbamazine	100 mg three times a day for 144 days	Probable cure	
12	Ms	thiabendazole	500 mg three times a day for 6 days	No cure	[33]
		
		diethylcarbamazine	100 mg two times a day	Probable cure	
13	NS and Ms	thiabendazole	50 mg/Kg of body weight a day for 5 weeks	No cure	[15]
	
		levamisole	50 mg daily for 10 days	No cure	
			150 mg a week for 3 months	Probable cure	
14	CR	levamisole	2.5 mg/Kg of body weight a day for 15 days	No follow-up	[13]
		and			
		praziquantel	15 mg/Kg of body weight (single dose)		
15	CR	thiabendazole	25 mg/Kg of body weight once a day for 10 days	No cure	[25]
		
		levamisole	150 mg once a day for 10 days	No cure	
		
		albendazole	400 mg once a day for 36 days	Probable cure	
16	NS	thiabendazole	30 mg/Kg of body weight a day for 3 days (poorly tolerated)		[11]
				No cure	
			15 mg/Kg a day for 6 days		
		mebendazole	200 mg/day for 4 days	No cure	
		levamisole	150 mg 3 times daily for 8 days	Probable cure	
			150 mg twice daily on 3 days of each week for the following 12 weeks		
17	ME, CNS	Associations:			[13]
		mebendazole thiabendazole	100 mg twice a day for 3 days	No cure	
			50 mg/Kg of body weight a day for 2 days		
		levamisole cambendazole	2.5 mg/Kg of body weight a day for 30 days 36 mg a day for 20 days	?	
18	Ms, TB and CNS	cambendazole	Four cycles of 30 mg/Kg of body weight a day for five days, repeated after a 10-day interval	No cure	[8]
		levamisole	150 mg por daily for 10 days, then 150 mg once a week for 3 months	No cure	
		ivermectin	Two cycles of four doses of 0.2 mg/Kg of body weight at weekly intervals, followed by a month without therapy; monthly doses thereafter for 6 months	Probable cure	
19	CR	ivermectin	300 μg/Kg of body weight at weekly intervals for 12 weeks	Probable cure	[30]
20	NS, Ms and ME	ivermectin	200 μg/Kg of body weight for 1 week	No follow-up	[7]
		and			
		thiabendazole	1 tab a day for 3 days, repeating after 15 days		

CR = cervical region, NS = nasal sinuses, Ms = mastoid, ME = middle ear, TS = tonsil, TB = temporal bone, SMR = Sub Maxillary Region, CNS = central nervous system.

ivermectin (*in vitro*) at a concentration of 200 μg per liter of 1% formalin, applied for 28 days, did not prevent embryogenesis or devitalize larvae inside the eggs of *L*. *minor* [47]. At a dose of 200 μg/Kg of body weight, the drug was ineffective on both third stage migratory larvae and third stage encysted larvae in infected mice. However, *in vivo*, at a dosage of 200 μg/Kg, it devitalized fourth stage larvae, arresting their development into adult worms in experimentally infected cats [48]. Levamisole hydrochloride at a concentration of 0.075 mg/Kg was ineffective against both third stage migratory larvae and third stage encysted larvae in infected mice [49].

### Social impact and prevention of the infection

Lagochilascariasis transmission is related to socioeconomic factors. Infected individuals usually live in rural areas. Government settlement projects in some Brazilian states have attracted individuals who, in their search for better work opportunities, move close to densely forested areas, where they live in poor sanitary conditions and become infected. Similarly to most tropical diseases, lagochilascariasis is a disease of poverty, mainly affecting populations with the lowest income [5]. Infected individuals generally live in precarious conditions in shanty houses or in dwellings at the edge of dense woodland areas and feed on the meat of wild animals such as armadillo, guinea pig, agouti, paca, wild boar, tortoise, and other animals [5,13,21]. Lagochilascariasis is not listed among the neglected diseases, but it fits this description perfectly. Like other neglected diseases, the drugs available to treat lagochilascariasis are very old.

Considering all the available research data on lagochilascariasis, it is clear that the inactivation of *L*. *minor* infecting larva is the main measure to prevent infection. Therefore, meat from wild animals, especially from rodents (guinea pigs and agouti) should be cooked at 100 ^o^C for 10 minutes or frozen at −20 ^o^C for 15 days before it is prepared for human consumption [5].

Lagochilascariasis is a zoonotic disease that does not represent a public health risk in any of the countries where it has been reported. Therefore, proposals for preventive sanitary measures to eradicate lagochilascariasis would be utopian and impractical, especially given the social and public health deficiencies in Neotropical countries, including Brazil. Nevertheless, the fact that a recent paper described the first report of *Lagochilascaris* eggs in a public park in Southern Brazil [50] is an indication that the disease is probably underrated.

**Key learning points**

- Lagochilascariasis is mostly a chronic human disease that can persist for several years, in which the parasite burrows into the subcutaneous tissues of the neck, paranasal sinuses, and mastoid. Other localizations of the parasite are the central nervous system, lungs, sacral region, eyeballs, and dental alveoli.
- The occurrence of auto-infecting cycles has been reported in both humans and cats. The parasite exhibits a remarkable ability to migrate through the tissues of its hosts, destroying even bone tissue.

**Top five papers**

1. Sprent JF. Speciation and development in the genus Lagochilascaris. *Parasitology*. 1971;62(1):71–112.
2. Campos DMB, Freire Filha LG, Vieira MA, Paçô JM, Maia MA. Experimental life cycle of Lagochilascaris minor Leiper, 1909. *Rev Inst Med Trop São Paulo*. 1992;34(4):277–87.
3. Campos DMB, Barbosa AP. Lagochilascaris. In: Neves DP, Melo AL, Linardi PM, Vitor RWA, editors. *Parasitologia humana*. 13aed. São Paulo: Atheneu; 2016. p.514-23.
4. Leão RNQ, Fraiha-Neto H, Dias LB. Lagochilascaríase. In: Veronesi R, Focaccia R, editors, *Tratado de Infectologia*. 5a. ed. São Paulo: Atheneu, 2015. P.2107-11
5. Paçô JM, Campos DMB. Lagochilascaris minor Leiper, 1909: Nove décadas de revisão bibliográfica. *Rev Patol Trop*. 1998;27(1):11–34.
